# 原发性非小细胞肺癌中线粒体DNA含量变化的研究

**DOI:** 10.3779/j.issn.1009-3419.2011.02.07

**Published:** 2011-02-20

**Authors:** 红梅 王, 纪刚 戴

**Affiliations:** 400037 重庆，第三军医大学新桥医院胸外科 Department of Thoracic Surgery, Xinqiao Hospital, Chongqing 400037, China

**Keywords:** 线粒体DNA, 肺肿瘤, 突变, mtDNA, Lung neoplasms, Mutation

## Abstract

**背景与目的:**

已有的研究表明：线粒体DNA（mitochondrial DNA, mtDNA）突变和拷贝数的改变和肿瘤有着密切联系；大多数实体性肿瘤中mtDNA拷贝数有明显降低。本研究旨在探讨线粒体基因组含量的改变和原发性非小细胞肺癌（non-small cell lung cancer, NSCLC）的关系。

**方法:**

通过实时荧光定量PCR，对肺癌及相对应的癌旁肺组织mtDNA的含量进行精确定量（拷贝数/细胞）。

**结果:**

肺癌组织mtDNA的平均拷贝数/细胞为395±125，而相对应的正常肺组织为733±196，前者明显低于后者（*P* < 0.001）。肺癌mtDNA含量的改变与患者性别、年龄、是否吸烟及肿瘤的病理类型无关（*P* > 0.05）。

**结论:**

mtDNA含量的改变与NSCLC的发生发展密切相关，同时也可能影响其治疗和预后。

## Abstract (en)

**Background and objective:**

It has been proven that the mitochondrial DNA (mtDNA) mutations and content change were associated with increasing risk of tumorigenesis. MtDNA content is significantly reduced in most substantive tumors. The aim of this study is to demonstrate whether mtDNA content is positively associated with non-small cell lung cancer (NSCLC) risk.

**Methods:**

MtDNA content in 37 matched lung carcinoma and histologically adjacent normal lung tissue samples from patients were analyzed by fluorogenic 5-nuclease real-time PCR techniques.

**Results:**

The mean copy number of mtDNA in lung carcinoma tissue samples was statistically lower than that in adjacent histologically normal lung tissue samples (*P* < 0.001).

**Conclusion:**

The change of mtDNA content may play an important role in NSCLC.

线粒体是真核细胞的重要细胞器，作为细胞的“动力工厂”，它在细胞的能量代谢和氧自由基生成过程中扮演重要角色。同时，线粒体也是细胞凋亡调控的关键元件，这些细胞器收集并处理有关细胞代谢和信号转导级联各方面的信号，决定着细胞的命运，参与了细胞死亡的执行过程。能量代谢模式的转换是癌细胞的重要特征。细胞在癌变过程中，将线粒体关闭，能量来源转换为糖原酵解模式，同时线粒体启动异常细胞凋亡的作用消失，这些细胞就可能“永生化”及癌变。线粒体DNA（mitochondrial DNA, mtDNA）含量（即拷贝数）及氧化磷酸化相关酶活性的降低是肿瘤能量代谢发生改变的主要原因。为了解非小细胞肺癌（non-small cell lung cancer, NSCLC）组织中mtDNA的拷贝数是否发生改变并探讨mtDNA拷贝数改变与NSCLC的相关性，本研究通过两个单独的实时荧光定量PCR对肺癌及相对应的癌旁组织mtDNA的含量进行了精确定量（拷贝数/细胞）。

## 1 材料与方法

### 1.1 病例资料

所用37例肺癌组织和37例癌旁肺组织均取自第三军医大学附属新桥医院2006年-2008年外科手术患者，均经病理确诊。癌旁肺组织标本为同一患者病灶远端5 cm以上的正常肺组织，均经组织学诊断，证实其没有癌细胞。本组患者男性28例，女性9例，吸烟及曾经吸烟者24例（吸烟者：每天吸烟10支以上且吸烟时间超过1年。本组中包括曾经吸烟而现戒烟1年以上者9例），不吸烟者13例。患者年龄30岁-75岁，中位年龄为55岁。组织学病理分型情况为：鳞癌23例，腺癌14例。

### 1.2 组织全基因组DNA提取

采用酚、氯仿、异戊醇抽提法提取全基因组DNA（包括mtDNA）。所得DNA溶于TE buffer（10 mM Tris±HCl, 1 mM EDTA, pH8.0），并用紫外分光光度计测定其波长260 nm时的吸光度（OD_260_ nm）值，计算DNA浓度，-20 ℃保存备用。

### 1.3 PCR引物及TaqMan探针

所有引物由上海基康生物技术有限公司合成、标记。本实验中应用的荧光检测物质有FAM（6-Carboxy-fluorescein）和TAMRA（6-Carboxytetramethy-rhodamine），其中FAM作为报告基因（Reporter），标记在TaqMan探针的5’。TAMRA作为熄灭基因（Q uencdher），标记在TaqMan探针的3’。TaqMan探针与目的基因的结合部位在两个引物之间。用于扩增人mtDNA D-loop *HV1*（Hipervariable region 1, HV1）和核*β*-*globin*基因的引物及TaqMan探针见表 1。

**1:** 引物序列和TaqMan探针 Primer and TaqMan probe sequence and length of PCR product

Target	Primer and probe name	Sequence (5’ -3’)	Length of PCR product (bp)
	Probe	CTCCTGAGGAGAAGTCTGCT	
*β*-globin	Forward primer	ACACAACTGTGTTCACTAGC	110
	Reverse primer	CAACTTCATCCACGTTCACC	
	Probe	CTCCCCATGCTTACAAGCAAGTACAGCAAT	
HV1	Forward primer	TTGCACGGTACCATAAATACTTGAC	128
	Reverse primer	GAGTTGCAGTTGATGTGTGATAGTTG	

### 1.4 线粒体HV1和核*β*-*globin*基因实时荧光定量PCR检测

#### 1.4.1 实时荧光定量PCR标准品的制备

##### 1.4.1.1 *HV1*和*β*-*globin*基因片段的PCR扩增

PCR反应体系为25 μL，终浓度为：Taq酶2 U，MgCl_2_ 2.5 mmol/L，dNTP各200 μmol/L，引物各0.5 μmol/L，模板DNA约100 ng。反应条件：94 ℃预变性5 min，其余40个循环94 ℃、30 s，55 ℃、30 s，72 ℃、1 min，最后72 ℃延伸10 min。

##### 1.4.1.2 PCR扩增产物的克隆

用TaKaRa公司的试剂盒对PCR样品进行纯化后，分别将128 bp HV1和110 bp β-globin扩增产物克隆至PMD18-T载体（TaKaRa公司），筛选含有插入片段的载体，以JM109感受态细胞进行转化。用质粒提取试剂盒（QIAGEN公司）提取质粒。

##### 1.4.1.3 质粒的鉴定与测序

将部分所提取的质粒经过PCR和序列测定进行鉴定，测序反应由上海基康生物技术有限公司完成。

##### 1.4.1.4 质粒浓度测定及拷贝数换算

紫外分光光度计检测其OD_260_值。质粒浓度（ng/μL）=OD_260_×50 μg/mL×稀释终体积（mL）×1, 000/稀释时加入的原始溶液体积（μL）。质粒拷贝数（Copies/μL）=质粒浓度（ng/μL）×6.02×10^14^/660×碱基数（载体+插入片段）。

##### 1.4.1.5 标准品的梯度稀释

以无菌去离子水10倍比梯度稀释HV1和β-globin质粒分别获得浓度为1×10^3^-1×10^7^拷贝/μL的制备定量PCR标准曲线的模板。

#### 1.4.2 实时荧光定量PCR检测

##### 1.4.2.1 PCR反应

25 μL体系中含ABI TaqMan 1×PCR Master mix、3.5 mmol/L MgCl_2_、200 μmol/L dNTP、引物各0.5 μmol/L、荧光探针50 nmol/L、模板各2.0 μL。每次检测设置3个NTC（no template control）。每个样品进行3个平行样实验。

##### 1.4.2.2 PCR反应条件

50 ℃、2 min，95 ℃、10 min；然后95 ℃、15 s，60 ℃、30 s，共50个循环。TaqMan PCR Core试剂盒、光学PCR管、7700型PCR仪均为PE公司产品。

##### 1.4.2.3 HV1和β-globin标准曲线绘制

将不同梯度浓度的标准品，与待测样品同时进行定量PCR扩增，绘制标准曲线。

##### 1.4.2.4 mtDNA拷贝数的检测

通过两个单独的实时荧光定量PCR分别对待测样本中线粒体*HV1*和核*β*-*globin*基因进行定量。以mtDNA HV1和核单拷贝基因*β*-*globin*分别作为线粒体和核DNA拷贝数的标记。mtDNA的含量，即相对拷贝数/二倍体核基因组（diploid nuclear genome）=2× HV1/β-globin^[1]^。

### 1.5 统计学方法

计量资料以Mean±SD表示，使用SPSS 11.0统计学分析软件进行t检验和线性回归分析。*P*＜0.05为差异具有统计学意义。

## 2 结果

### 2.1 克隆质粒的鉴定

提取经筛选的用于制作标准曲线模板的质粒，经过PCR及序列测定证实， *HV1*和*β*-*globin*基因PCR片段已分别连接至PMD18-T载体上，并用所建立的实时荧光定量PCR检测，可见有明显的荧光信号。

### 2.2 标准曲线的制备

根据不同浓度的标准品测得的荧光信号到达设定的域值时所经历的循环数（threshold cycle, Ct）值绘制标准曲线（图 1）。不同浓度标准定量的模板与Ct值呈直线相关。*HV1*和*β*-*globin*基因定量PCR标准曲线的相关系数分别为0.998和0.999，斜率分别为-3.159和-3.400。

**1:**
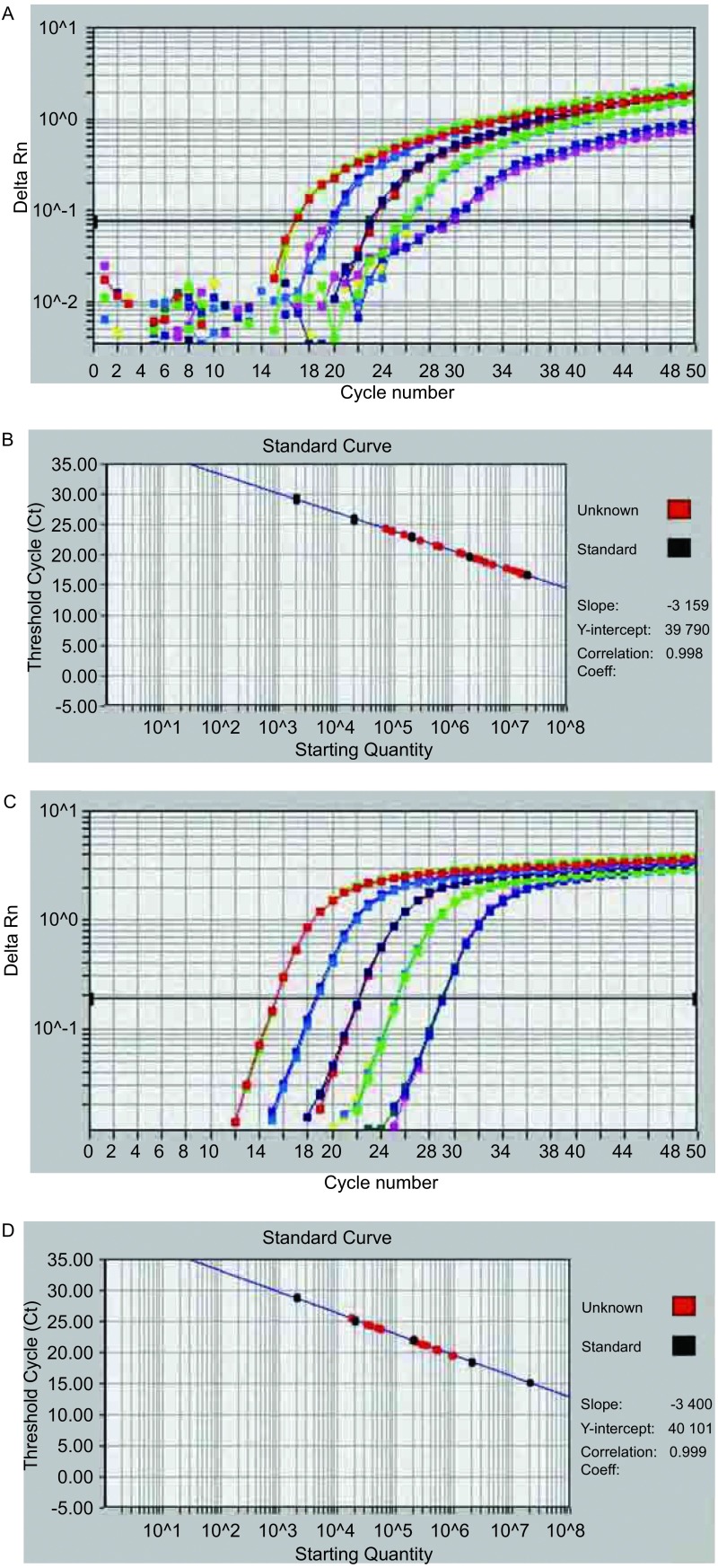
mtDNA HV1（图 1A，图 1B）和核基因（图 1C，图 1D）实时荧光定量PCR动力曲线图和标准曲线 Amplification plots and standard curves of real-time PCR for Mitochondrial DNA HV1 (Fig 1A, Fig 1B) and nuclear *β*-globin gene (Fig 1C, Fig 1D)

### 2.3 肺癌mtDNA拷贝数的改变

为了解肺癌组织中mtDNA含量的改变，我们通过两个单独的实时荧光定量PCR，对肺癌及相对应的癌旁肺组织mtDNA含量进行了精确定量（拷贝数/细胞）。线性回归分析表明，肺组织mtDNA拷贝数和相对应的正常肺组织mtDNA拷贝数之间有相关性（*r*=0.589, *P*＜0.01）（图 2）。肺癌组织mtDNA的平均拷贝数/细胞为395±125，而相对应的正常肺组织为733 ±196，前者明显低于后者（*P*＜0.001）。

**2:**
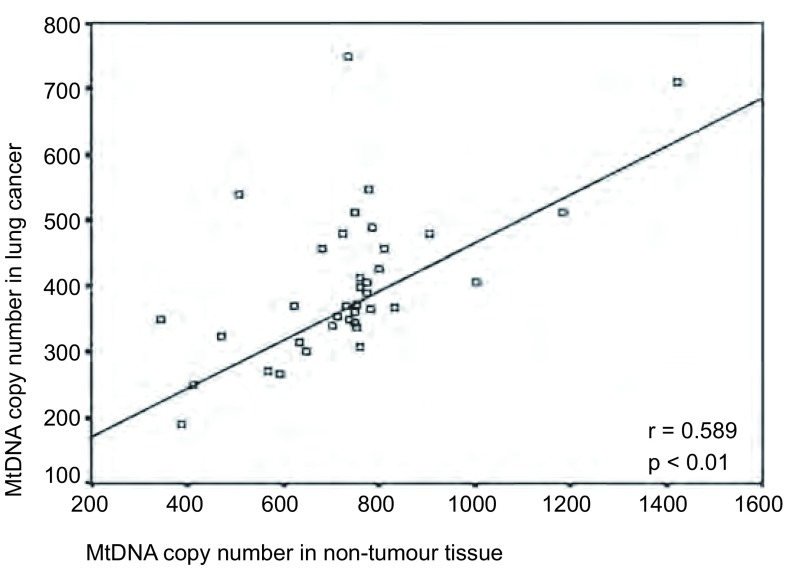
肺癌及相对应的癌旁肺组织mtDNA拷贝数散点图及线性相关分析 MtDNA copy number in lung cancinoma and adjacent nomal samples

### 2.4 肺癌mtDNA拷贝数改变和临床指标的相关性

根据患者性别、年龄、是否吸烟、病理类型，我们将37例样本进行分组。统计结果显示，肺癌mtDNA拷贝数的改变与患者性别、年龄、是否吸烟及肿瘤的病理类型无关（*P*＞0.05）（表 2）。

**2:** 肺癌线粒体DNA拷贝数的改变和临床指标的关系 MtDNA copy number per nucleus in relation to clinicopathological variables in lung cancer

	*n*	MtDNA copy number	*P*
Gender			0.501
Female	9	419±82	
Male	28	396±123	
Age			0.306
≥55	19	429±129	
< 55	18	380±91	
Histological type			0.948
Squamous cell	23	388±111	
carcinoma			
Adenocarcinoma	14	427±119	
Smoking status			0.685
Smoker	24	393±114	
Non-smoker	13	419±116	

## 3 讨论

哺乳动物mtDNA是一全长为16.5 kb左右的双链闭环分子。它含有37个基因和一个非编码区（控制区），非编码区又称之为D环（displace loop, D-loop），其内有控制mtDNA转录和翻译的调节序列。人类mtDNA D-loop的起始和结束位置分别为mtDNA的16, 023和576核苷酸处，长度为1, 122 bp。mtDNA两条互补链的碱基组成呈非对称性。一条链称重链，富含G碱基，编码37个线粒体基因中的28个。另一条链称轻链，G碱基较少，编码其余的线粒体基因。与核不同的是，线粒体基因组为多拷贝基因组。体细胞中平均含有100个-500个线粒体，而每一个线粒体中又含有1个-15个mtDNA分子。呼吸链的正常组合和运转需要一个完整和功能性的线粒体基因组，而mtDNA功能的完成既依赖于每一个mtDNA分子结构的完整性，也同时依赖于细胞中mtDNA的拷贝数。

大多数实体性肿瘤中，能量代谢的改变和mtDNA含量有着密切的联系。Meierhofer等^[1]^研究表明肾癌组织中mtDNA含量和酶活性显著低于癌旁组织，并且两者的下调呈一致性。Mambo等^[2, 3]^发现80%的乳腺癌组织其mtDNA拷贝数低于相应的正常组织。研究^[4]^表明线粒体酶活性的降低与肿瘤的类型和分化程度密切相关。目前还在肝癌、结肠癌等多种肿瘤及肿瘤细胞株中发现存在有mtDNA含量的降低^[5]^。在本研究中，我们对NSCLC及相对应的癌旁正常肺组织mtDNA拷贝数进行了精确定量（拷贝数/细胞），结果发现，肺癌组织mtDNA的平均拷贝数/细胞低于相对应的癌旁正常肺组织。但我们并没有观察到mtDNA拷贝数的改变与患者性别、年龄、是否吸烟及肿瘤的病理类型之间的相关性。由此我们可以推论，mtDNA拷贝数量的改变与NSCLC的发生有密切关系，与患者性别、年龄、是否吸烟等因素无关；但NSCLC与小细胞肺癌之间是否有差异性则无法判断。这与Matthew等^[6]^的研究结果具有一致性。

研究表明肿瘤的发生与细胞凋亡异常密切相关，而线粒体途径是介导细胞凋亡的经典途径之一。一些环境因素的改变或者有害物质的入侵，可造成线粒体肿胀破裂或多个线粒体融合成一个大线粒体，影响呼吸链电子传递，造成细胞死亡。这些都表明线粒体在肿瘤的发生过程中具有重要作用。我们检测到肺癌组织中mtDNA的含量低于癌旁正常组织，表明mtDNA拷贝数降低可能导致肺癌细胞凋亡减少，从而促进了肺癌的生长、浸润和转移。

由于肿瘤细胞线粒体在分子、生化、代谢和遗传水平上明显区别于正常细胞，恶性肿瘤组织氧化抑制抑制酵解的现象减弱甚至消失，而代之以酵解抑制氧化，此即Crabtree效应。我们实验测得正常肺组织mtDNA平均拷贝数/细胞为733±196，低于骨骼肌细胞中的1, 811±546^[7]^和心肌细胞中的6, 970±920拷贝数/细胞^[8]^，这与肺组织细胞能量代谢和有氧ATP生产的需求低于骨骼肌和心肌细胞的特点是一致的。临床研究调查表明肺肿瘤的发生率远远大于骨骼肌和心肌肿瘤的发病率。由此我们可以推论，mtDNA含量低的组织细胞更易适应糖酵解供能的生长模式，从而发生肿瘤的几率更高。

研究^[9]^发现线粒体特异性的抑制剂处理和mtDNA的部分减损，可通过某个应激信号而诱导肺癌A549细胞系出现侵袭性肿瘤表型；mtDNA缺失细胞系HeLa rho0细胞对阿霉素和光动力性疗法（photodynamic therapy）诱导的细胞死亡具有极端的耐受性，而HeLa rho+细胞却非常敏感^[10-12]^；这表明mtDNA含量降低不仅与恶性肿瘤的发生和转移有关，还关系到肿瘤的治疗和预后，NSCLC中mtDNA含量较低可能是其化疗敏感性差的原因之一。

目前我们的研究结果表明mtDNA含量降低可能与NSCLC的发生、转移、治疗和预后具有密切关系。但引起这一变化的原因和具体机制还不清楚。我们将进一步探索研究，以期能为NSCLC的治疗提供更多理论基础。
